# Tuning the Properties of PNIPAm-Based Hydrogel Scaffolds for Cartilage Tissue Engineering

**DOI:** 10.3390/polym13183154

**Published:** 2021-09-17

**Authors:** Md Mohosin Rana, Hector De la Hoz Siegler

**Affiliations:** 1 Biomedical Engineering Graduate Program, Schulich School of Engineering, University of Calgary, Calgary, AB T2N 1N4, Canada; mdmohosin.rana@ucalgary.ca; 2 Department of Chemical and Petroleum Engineering, Schulich School of Engineering, University of Calgary, Calgary, AB T2N 1N4, Canada

**Keywords:** PNIPAm, synthesis-solvent, crosslinking, cartilage, tissue engineering, scaffold

## Abstract

Poly(N-isopropylacrylamide) (PNIPAm) is a three-dimensional (3D) crosslinked polymer that can interact with human cells and play an important role in the development of tissue morphogenesis in both in vitro and in vivo conditions. PNIPAm-based scaffolds possess many desirable structural and physical properties required for tissue regeneration, but insufficient mechanical strength, biocompatibility, and biomimicry for tissue development remain obstacles for their application in tissue engineering. The structural integrity and physical properties of the hydrogels depend on the crosslinks formed between polymer chains during synthesis. A variety of design variables including crosslinker content, the combination of natural and synthetic polymers, and solvent type have been explored over the past decade to develop PNIPAm-based scaffolds with optimized properties suitable for tissue engineering applications. These design parameters have been implemented to provide hydrogel scaffolds with dynamic and spatially patterned cues that mimic the biological environment and guide the required cellular functions for cartilage tissue regeneration. The current advances on tuning the properties of PNIPAm-based scaffolds were searched for on Google Scholar, PubMed, and Web of Science. This review provides a comprehensive overview of the scaffolding properties of PNIPAm-based hydrogels and the effects of synthesis-solvent and crosslinking density on tuning these properties. Finally, the challenges and perspectives of considering these two design variables for developing PNIPAm-based scaffolds are outlined.

## 1. Background

Bone, cartilage, and skeletal muscle can be severely injured by repeated overuse [1]. Due to their complex microstructural composition, severe injury to these tissues cannot heal naturally [2]. To heal these injuries, current treatment methods include autologous and allogeneic grafting [3]. Autografts are active tissue parts coming from the same patient. Hence, autografting shows a higher rate of success and speeds up the healing process. Although it is considered the gold standard of treatment [4], this approach has some limitations, including limited availability of donor tissue graft, multiple surgeries required to collect and separate the grafts, and higher chance of morbidity and associated complications at the donor site [4]. Allografting, on the other hand, uses the tissue of a deceased donor for transplanting into the patient [5]. This method has some advantages over autograft including no donor site morbidity, shorter surgical time and smaller incisions, and higher availability for transplant [6]. Major drawbacks include potential immune rejection after grafting and a higher risk of disease transmission. Tissue engineering techniques are being developed to overcome the limitations of both autograft and allograft methods.

Tissue engineering is an interdisciplinary and rapidly growing field in life sciences involving cell biology, biochemistry, clinical medicine, materials science, cell–material interactions, and surface characterization [7]. The main goal of this field is to regenerate new biological tissues to replace previously damaged or diseased organs or tissues. Tissue engineering typically involves the isolation of targeted cells (progenitor or stem cells) from living tissues and harvesting in vitro. The isolated cells are then seeded and expanded on three-dimensional (3D) scaffolds (natural or synthetic materials). These scaffolds mimic the natural extracellular matrices (ECM) by providing support for cell function, adhesion, and transplantation [8]. The key roles of scaffolds in tissue engineering are: deliver the seeded cells to the desired site; stimulate cell–material interactions; induce cell adhesion; and allow sufficient transport of nutrients and growth factors to support cell survival, proliferation, and differentiation [9]. All these roles of scaffolds help to control the structure and function of the engineered tissue. The cell-loaded scaffolds are then transplanted into the patient’s body in any of the following ways: (1) direct injection via needle or other minimally invasive delivery process and (2) implantation of the regenerated tissue at the desired site using traditional surgery [10]. Finally, the scaffold degrades gradually as the tissue develops and the damaged tissue is regenerated.

Among the different types of scaffolds, polymer hydrogels have attracted significant attention in tissue engineering due to their similarity to the cellular microenvironment and their tunable physicochemical properties [11]. Hydrogels play an important role in tissue engineering due to their distinctive characteristics such as hydrophilicity, super-absorbency (up to 1,000-fold compared to their dry weight), biocompatibility, biodegradability, porosity, softness, and viscoelastic behavior resembling that of the natural tissue [12]. Hydrogels are 3D network structures of highly hydrophilic regions formed via physical or chemical crosslinking. Due to their crosslinked 3D structure, they can encapsulate cells in a homogeneous manner, and enhance cell–cell and cell–ECM interactions by providing a 3D microenvironment similar to the native ECM [11].

An important class of hydrogels is stimuli-responsive, or smart, hydrogels [13]. In response to external stimuli (e.g., temperature, pH, light, electric field, or ionic strength) these hydrogels undergo a change in volume or a sol–gel phase change in a reversible manner [14]. This unique feature effectively mimics the sensitivity of biomolecules in the biological environment. Temperature-responsive or thermoresponsive hydrogels are the most widely used smart hydrogels due to their capacity for reversible phase transition by a slight change of temperature [15]. The topographical and physical properties of hydrogel scaffolds enable the control of biological functions (e.g., cell attachment/detachment, protein absorption, and cell differentiation) [16]. The phase transition of thermoresponsive hydrogels provides hydrophobic/hydrophilic surfaces for cell attachment/detachment and also regulates cell proliferation [16,17]. Thermoresponsive hydrogels can be classified into two categories, i.e., lower critical solution temperature (LCST) hydrogels that can be hydrated and swell at lower temperatures, and upper critical solution temperature (UCST) hydrogels that can swell and hydrate at higher temperatures [18]. Poly(N-isopropylacrylamide) or PNIPAm is a widely studied LCST polymer because it has an LCST of approximately 32–33 °C, which is close to body temperature (37 °C) [19]. That means PNIPAm hydrogels can form a sol state at room temperature and transform into their gel state at close to physiological temperature [15]. This property makes PNIPAm hydrogels excellent candidates as potential scaffolds for tissue engineering. For instance, PNIPAm hydrogels can be applied as an injectable formulation with stem cells into the body where they can act as extracellular support [10], helping the cells to maintain normal physiological activity and promoting cell proliferation and differentiation to repair damaged tissue [20]. PNIPAm hydrogels can also be used as a 3D scaffold for in vitro regeneration of tissue which can later be transplanted via minimally invasive surgery [10]. Despite PNIPAm scaffolds’ superior properties such as tunable structures, thermosensitivity and low toxicity, there are some drawbacks including poor biocompatibility and weak mechanical properties that hinder their broader applicability [15].

The structure and physicochemical properties of PNIPAm scaffolds useful for cartilage tissue engineering can be controlled by selecting different synthesis-solvents, crosslinking methods, different biomaterials, and fabrication strategies [21,22]. The objective of this review is to provide a comprehensive and critical assessment of recent advances in the tuning of scaffolding properties of PNIPAm hydrogels, with special focus on the role played by polymer synthesis conditions. First, requirements for cartilage regeneration and the applicability of PNIPAm in cartilage repair are explored. Then, the effects of synthesis-solvents and crosslinking methods, as well as crosslinking density, on the structural changes and physicochemical properties are described in detail. Finally, the challenges of selecting synthesis-solvents and the potential for replacing them with more sustainable and less toxic alternatives are discussed.

## 2. Scaffolds for Cartilage Regeneration

It is crucial to understand the complex structure of the articular cartilage (AC) before developing a mimicking construct to repair damaged tissue. Articular cartilage is an elastic tissue that consists of spheroid chondrocyte cells (2% of the total volume of the AC) protected by the surrounding ECM [23]. The solid phase of the AC is porous and permeable, while the main part of the fluid phase is water containing inorganic ions such as sodium, potassium, and chloride [24]. The ECM, which provides support and protection for the chondrocytes, is mainly composed of water, collagens (type II), proteoglycans, and non-collagenous glycoproteins [23,24]. The main function of the AC is to transmit loads to the related subchondral bone and absorb impact forces, resulting in low-friction gliding between the surfaces of the joints [24]. Damage in the AC of joints can occur due to trauma, unhealthy lifestyle, age, and various diseases including autoimmune disorders [25]. This leads to an excoriation of the cartilage surface and loss of elasticity and resistance to friction [26]. As the cartilage is avascular and aneural, it is incapable of transferring nutrients to cells, and thus not able to heal naturally [27]. Although there are several treatment methods currently available, none of them is successful in recreating native cartilage [28]. Tissue regeneration using advanced scaffolds, growth factors and nutrients, and progenitor or stem cells, provides an alternative treatment option for effectively recreating native cartilage.

**Figure 1:**
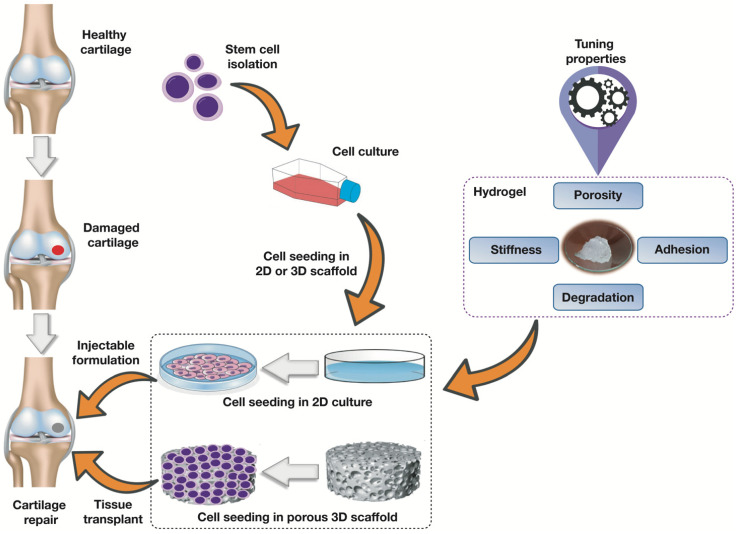
Schematic diagram showing stem cell isolation and tissue regeneration in hydrogel scaffolds with tunable properties for cartilage tissue regeneration.

Cartilage tissue regeneration requires scaffolds capable of providing a proper environment to enhance cell adhesion, migration, and proliferation [29]. There are two approaches to regenerate cartilage onto scaffolds, namely 2D and 3D scaffolds (Figure 1) [30]. The cell response in the microenvironment of 3D scaffolds differs from that in the conventional 2D cell culture system [30]. The 3D design is more effective, as it helps to prevent the dedifferentiation of chondrocytes into fibroblast-like cells [31,32]. In the case of 2D scaffolds (flat scaffold surface), chondrocytes may lose their ability to generate proteins that are required for the formation of cartilage [33]. For successful cartilage tissue repair, PNIPAm-based materials used as scaffolds need to support the survival and differentiation of progenitor or stem cells used. This can be confirmed by analyzing the basic physical properties of PNIPAm-based hydrogels. Physical properties of hydrogel scaffolds such as stiffness and mechanical strength, porosity, adhesion, and degradability are critical in cartilage tissue engineering (Table 1). These properties can be tuned at different levels to fulfil the basic requirements for cartilage regeneration.

### 2.1. Porosity

The microstructure of hydrogels is a critical factor in designing scaffolds for cartilage tissue regeneration, as it can affect the cells’ activity and functions [11]. Generally, the structure of bulk hydrogels is a dense polymer network with nano-sized pores [34]. Such a nano-porous structure is too small to facilitate nutrient diffusion, cell migration, and proliferation [35]; a microporous structure is required instead [21]. The effects of pore structure and pore sizes have been studied for tissue engineering applications [36]. Scaffolds with a 300 μm mean pore size exhibited higher chondrogenic gene expression than scaffolds with a smaller mean pore size of approximately 100 μm [37]; chondrocytes cultured in microporous hydrogel scaffolds showed high proliferation and promoted cell migration into the microporous cavities [21]. Hence, the engineering of porous scaffolds is important to promote chondrocyte cell differentiation and successful articular cartilage tissue engineering.

### 2.2. Stiffness and Mechanical Strength

Conventionally synthesized hydrogels are breakable, which in turn reduces their stability; hence, these materials cannot be used without modifications for cartilage tissue engineering applications. Stiffness and mechanical strength are important for maintaining the stability of the scaffolds and to ensure effective cell activities and fates [38], as the mechanical signals at the microscopic level influence chondrogenic, vascular, and neural cell differentiation [39]. Thus, cell morphology and chondrogenic gene expression can be regulated by controlling the stiffness of hydrogel scaffolds. In chondrogenic cell differentiation, hydrogel scaffolds with lower stiffness lead to higher mRNA levels of chondrogenic markers Col2α1, Agc, and Sox9 [40]. Although different hydrogel scaffolds have been developed to support healthy cartilage formation, mimicking the mechanical properties and gaining the resiliency of articular cartilage is still a matter of concern in developing scaffolds for cartilage tissue engineering.

### 2.3. Cell Adhesibility

Cell adhesion onto the hydrogel scaffolds is important as it can greatly affect cell behaviors such as spreading (on the scaffold surface), proliferation, and differentiation [41]. Cell adhesibility can be tuned by introducing bioactive peptides (e.g., arginine-glycine-aspartate or RGD peptides) onto the hydrogel surface [42], as cell adhesion ligands or peptides are key biochemical components in the ECM [43]. Cell adhesion to the scaffold is mediated by the specific interactions of cell surface receptors (e.g., integrin) with the scaffold to maintain cell functions and viability [44]. Integrin receptors recognize RGD as the primary cell attachment site [45]. Therefore, it is reported that RGD peptides in hydrogel scaffolds trigger the chondrogenic gene expression when the scaffolds are loaded with dynamic mechanical forces [46]. Moreover, the crosslinking and mechanical properties of the hydrogel scaffolds also significantly influence cell adhesion, which in turn regulates cell proliferation and migration in the scaffolds’ microenvironment [47]. Therefore, the effects of crosslinking on cellular adhesion, proliferation, and migration are very crucial to consider when designing hydrogel scaffolds for cartilage regeneration.

**Table 1:** Key scaffolding properties of hydrogels used in cartilage tissue engineering.

Property	Features and Performance	References
Porosity	- higher porosity induces chondrocyte migration and proliferation - high porosity enhances cell spreading and type II collagen production - larger pore sizes improve gene expression and ECM secretion - 300 μm pore size is ideal for chondrogenic gene expression	[48,49,50]
Mechanical Strength	- higher mechanical strength enhances cartilage regeneration - scaffolds with strong mechanical properties (i.e., elastic modulus) accelerate cell migration and spreading and cell differentiation	[51]
Stiffness	- lower stiffness gives higher mRNA levels of chondrogenic markers (Col2α1, Sox9, and Agc) - higher stiffness yields a higher percentage of cells with chondrocytic morphology - higher elastic modulus promotes hypertrophic differentiation of chondrogenic cells	[40,52,53]
Adhesion	- higher adhesion stimulates chondrogenic cell spreading, proliferation, and differentiation - adhesive ligands trigger chondrogenic gene expression	[41,54]
Degradation	- lower degradation of hydrogel scaffold is good for chondrogenesis of stem cells - differentiation of mesenchymal stem cells (MSCs) is regulated by degradation-mediated cellular traction - hydrolytically stable hydrogels show a higher level of chondrogenic marker gene expression and lower level of hypertrophic genes	[55,56,57]

### 2.4. Degradation

In tissue engineering applications, hydrogel scaffolds’ degradability is a key concern. Once the targeted tissue regenerates, the scaffold must degrade so that the tissue construct can be retrieved (in vitro) and/or be adapted into the physiological environment (in vivo) [58]. Degradable hydrogel scaffolds are broken down after a certain time into smaller polymer blocks, which are small enough to be egested from the body. Covalently crosslinked hydrogels usually undergo degradation via enzymatic hydrolysis, ester hydrolysis, or photolytic cleavage of the polymer chains [59]. Hydrogel scaffolds can be designed based on these degradation mechanisms to make the scaffold a better temporary support with good biodegradability and desirable degradation rates. Such degradable scaffolds will be gradually degraded and replaced with the regenerating tissues. Although degradation of hydrogel scaffolds is a chemical process, it can act as a dynamic physical stimulus that mimics the native ECM [60]. Hence, such a process can affect cell spreading, migration, proliferation, and differentiation, thus impacting tissue regeneration. The rate of degradation can be tuned by altering the ratio of crosslinker to monomer during synthesis [61]. In PNIPAm-based copolymeric scaffold synthesis, degradation time can be regulated by tuning the ratio of PNIPAm polymer to another polymer [62]. As the stiffness or mechanical strength of the scaffolds typically decreases as the hydrogels degrade, it becomes impossible to assert which factors affect cell behavior and tissue regeneration [21]. Consequently, it is highly desirable to design hydrogel scaffolds able to degrade without changing their mechanical properties.

## 3. Application of PNIPAm-Based Scaffolds in Cartilage Tissue Engineering

PNIPAm is an amphiphilic thermoresponsive polymer that contains both hydrophilic (amide moiety) and hydrophobic (isopropyl moiety) regions in its structure [63]. Their amphiphilic nature is responsible for the phase changes observed in PNIPAm as a function of temperature. Above the LCST, PNIPAm hydrogels become hydrophobic and provide an adhesive surface for cell attachment and proliferation; when the temperature decreases below the LCST, the cells spontaneously detach [64]. This behaviour makes PNIPAm and its copolymers attractive candidates in tissue engineering and other biomedical applications. However, poor mechanical properties and limited biocompatibility are the two major drawbacks that limit their use in tissue engineering applications such as cartilage regeneration [15].

To improve PNIPAm’s mechanical properties, copolymerization with other monomers has been studied [65]. Poly(N-isopropylacrylamide-co-acrylic acid) (PNIPAm-co-AAc) hydrogels were found to be more stable than PNIPAm hydrogels and showed a lower extensive capacity change [66,67]. Hence, chondrocytes seeded in PNIPAm-co-AAc could proliferate and maintain their phenotype better than in PNIPAm scaffolds [67,68]. PNIPAm-co-AAc scaffolds promoted in vitro cell proliferation and the chondrogenesis of chondrocytes when growth factors (such as TGF-β3 and glucocorticoids), and other nutrients such as vitamin C were provided [68]. Despite the advanced mechanical properties of PNIPAm-co-AAc scaffolds, biocompatibility and immunogenicity of such hydrogels are still major issues [67]. Hence, more studies are required to better understand the biocompatibility of PNIPAm-co-AAc for in vivo tissue engineering applications.

Integration of biocompatible copolymers into a PNIPAm structure has also been explored to improve its mechanical properties while addressing biocompatibility or cytotoxicity issues [69]. A hybrid scaffold containing polyethylene glycol (PEG) and poly(ε-caprolactone) (PCL) microfibers with PNIPAm (PEG-b-PNIPAm) was developed to encapsulate human MSCs (hMSCs) [70]. Rather than cell encapsulation, cell attachment on the outside surface of such a hydrogel scaffold is a matter of concern due to the poor cell-adhesive properties of PCL that halt its wide applicability [71]. Kwon and Matsuda used a copolymer block of PNIPAm and PEG as thermoresponsive support for chondrocyte immobilization in cartilage tissue repair [72]. This 3D scaffold showed a minimal decrease in cell number with excellent cell viability and maintained the morphological characteristics of native chondrocytes. Detailed cell function and biodegradability studies are, however, required before using this hydrogel scaffold for cartilage and other tissue engineering applications, as the fragmented polymer microaggregates after degradation may be scavenged with a extended period of implantation [72]. Ma et al. developed PNIPAm hydrogels in conjugation with methacrylate-polylactide (MAPLA) and 2-hydroxymethyl methacrylate (HEMA) as an injectable solution [73]. This bioabsorbable thermoresponsive hydrogel showed no cytotoxicity with increased mechanical strength and lower resorption rates for several months. Despite these advantages, the inability of HEMA to support protein adsorption, cell attachment, and growth of mammalian cells are major outstanding issues [74,75]. Poor cell attachment and low interaction ability between MAPLA scaffolds and the surrounding tissue have also been observed, due to the poor hydrophilicity of PLA derivatives [76].

Chitin is the second most abundant natural polymer, procured from the exoskeleton of marine crustaceans, fungi, and insects [77,78]. Chitosan is a linear polysaccharide obtained from partial deacetylation of chitin [77]. Chitosan is broadly studied in tissue engineering due to its abundance, biocompatibility, biodegradability, non-toxicity, anti-microbial properties, and hydrophilicity [79]. Poor mechanical properties in wet conditions are, however, a major limitation [80]. Hence, chitosan is copolymerized with synthetic polymers such as PNIPAm to induce mechanical properties for tissue engineering applications [81]. In a study, a thermoresponsive 3D porous hydrogel scaffold containing PNIPAm-COOH and chitosan was prepared with an LCST of approximately 30 °C [82]. This scaffold exhibited good phenotypic morphology maintenance of the entrapped chondrocytes, triggered the initial cell–cell interactions, and preserved cell viability. The hMSCs encapsulated with chitosan-PNIPAm complex showed enhanced gene expression of Col II and aggrecan, and successful differentiation into chondrocytes in vivo [83]. These promising results confirmed the potential of chitosan-PNIPAm composite scaffolds for cartilage tissue engineering. Although the mechanical properties of chitosan-based hydrogel scaffolds have been improved over the years, the restricted solubility of chitosan is still a major issue [84]. Moreover, additional efforts are needed to tailor the pore morphology of chitosan-based scaffolds to the precise tissue requirements.

Hyaluronic acid (HA) or hyaluronan is a nonsulfated glycosaminoglycan (GAG) naturally found ubiquitously in the extracellular matrix (ECM) [85]. HA is an important structural element of the ECM, where it mediates cell migration and proliferation, wound repair, and matrix organization [86]. Strong hydrophilicity, high water absorption capacity, biocompatibility, and biodegradability make HA-based scaffolds promising candidates for tissue engineering [87]. Despite its advantages, HA alone exhibits poor mechanical properties with rapid degradation behavior [87]. Hence, HA has been crosslinked with other polymers to overcome these issues. In a previous report, an adipose tissue-derived stem cells (ADSCs)-encapsulated HA-crosslinked PNIPAm scaffold showed high viability, increased gene expression of chondrogenic markers and better in vivo hyaline cartilage formation [88]. A PNIPAm-co-AAc and HA composite scaffold also induced chondrocyte differentiation in the presence of TGF-β3 [89]. In a recent report, thermoresponsive hydrogels were fabricated from norbornene functionalized HA (NorHA) crosslinked with dithiol-terminated PNIPAm (DTPN). hMSCs adhered and proliferated successfully on the DTPN patterned surface of the hydrogel [90]. HA-based scaffolding materials still face some challenges. Absorption of proteins onto the implanted HA-based scaffolds is a major concern, as it might induce several degeneration effects [91]. In addition, the rapid degradation of HA-based composite scaffolds is still a matter of concern that can potentially be addressed by tuning the pore morphology of the composite scaffolds during synthesis [92].

In general, PNIPAm-based copolymer hydrogels appear to provide an appropriate milieu for in situ scaffolds for cartilage tissue engineering. Significant progress has been reported, as summarized here, in addressing the biocompatibility and mechanical properties of PNIPAm-based scaffolds. The following sections of this review explore more detailed approaches to carefully tune the properties of these hydrogel scaffolds.

## 4. Tuning Scaffolding Properties

### 4.1. Synthesis-Solvent Effects

PNIPAm hydrogels are typically synthesized via free radical polymerization. The synthesis-solvent used in polymerization acts as a chain transfer agent, affecting the termination of the growing chains and the reinitiation of growth in other chains [22]. The polarity of the synthesis-solvent plays a significant role in determining its reactivity [93]. Theoretical studies have indicated that polar solvents could have a faster chain termination rate than non-polar solvents [94]. This behavior has also been experimentally observed, as presented in recent reports [22,95].

**Figure 2:**
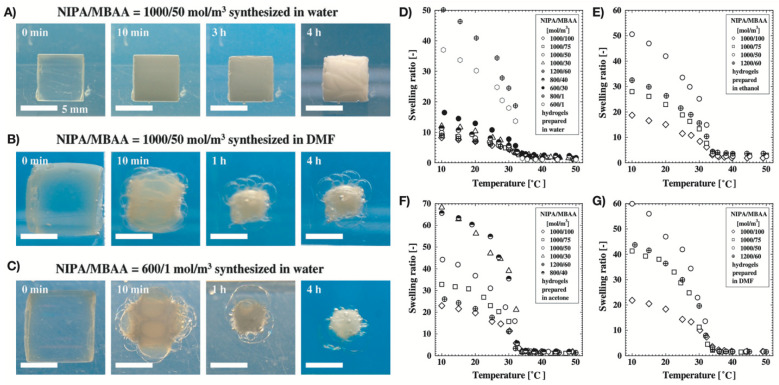
(**A***–***C**) Morphological changes of NIPA hydrogels after temperature changes between 10 °C and 50 °C, and (**D**–**G**) swelling ratio vs. temperature curves of the cylinder-shaped NIPA hydrogels in water, ethanol, acetone, and DMF, respectively. Reproduced with permission from Tokuyama et al. (2007) [96].

A broad range of studies have reported on how the synthesis-solvent affects the porosity, elasticity, and swelling behaviour of the synthesized polymers. Tokuyama et al. reported the synthesis of NIPA hydrogels copolymerized with N,N’-methylenebisacrylamide (MBAA) via free radical polymerization in four solvents: water, acetone, ethanol, and N,N-dimethylformamide (DMF) [96]. The swelling and elastic properties of NIPA indicated that these properties were affected by the type of synthesis solvent (Figure 2). At 10 °C, hydrogels synthesized in water had a smaller swelling volume and higher shear modulus than hydrogels synthesized in the other three amphiphilic solvents. The crosslinking network of the hydrogels was also affected by the synthesis-solvent. NIPA hydrogels synthesized in water had an inhomogeneous network structure due to the entanglement of polymer chains; in contrast, hydrogels synthesized in amphiphilic solvents were homogeneous due to the lower polymer concentration than the pre-gel solution. The conversion from monomers and MBAA to gel increased with an increase in MBAA and monomer concentrations in the pre-gel solution. In water, this conversion was higher than in other amphiphilic solvents. Additionally, the gelation was faster in water than that in amphiphilic solvents. The main reason behind this was the good reactivity of water with monomers and MBAA. Contrarily, the other solvents were poorly reactive to copolymerization into gels. Hence, NIPA hydrogel concentrations were lower in amphiphilic solvents than in water. A similar homogeneous network structure could be observed in the lightly crosslinked hydrogels synthesized in water. Moreover, the shear modulus of NIPA hydrogels synthesized in water was one order of magnitude higher than that of hydrogels synthesized in other amphiphilic solvents. In a subsequent study, Tokuyuma et al. investigated the structure of NIPA hydrogels polymerized in water and DMF solvent mixtures [97]. They found that the NIPA gels could have homogeneous/heterogeneous structures depending on the mole fraction of DMF (X_D_). Microgels synthesized in a solvent mixture with X_D_ = 0.25 had a porous structure that formed as the microgel aggregates phase-separated due to the cononsolvency. Due to the higher porosity, these microgels experienced very rapid shrinking in response to temperature change above the LCST, and vice versa.

**Figure 3:**
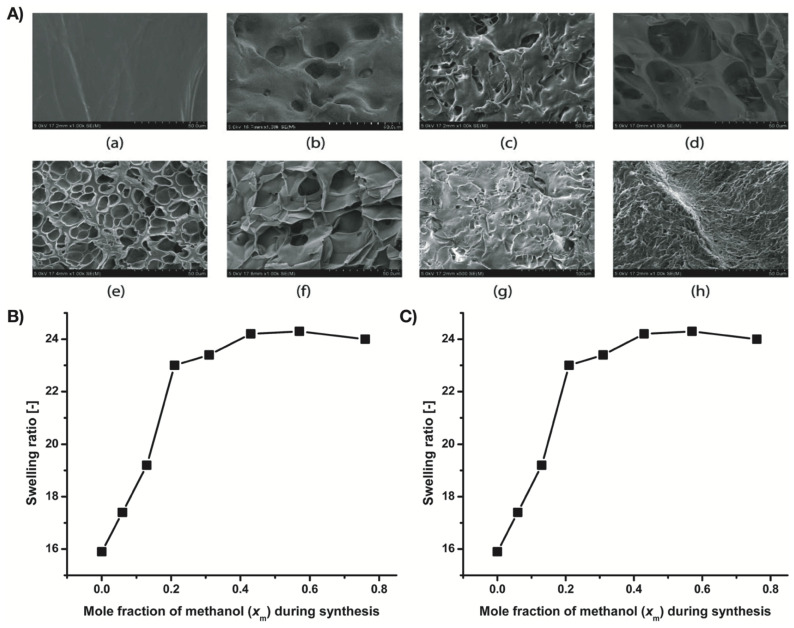
(**A**) SEM images of surface morphology of copolymer gels of NIPAM and NEAM: (a) X’_0_, (b) X_0_, (c) X_0.06_, (d) X_0.13_, (e) X_0.21_, (f) X_0.31_, (g) X_0.43_, and (h) X_0.57_, (**B**) Swelling ratio vs. solvent composition curve of the hydrogels at 20 °C, and (**C**) Young’s modulus vs. mole fraction of methanol curve of the hydrogels. Reproduced with permission from Wang et al. (2017) [99].

García and Cortés carried out the polymerization of acrylamide in different water/ethanol proportions to synthesize polyacrylamide (PAAm) [98]. From the swelling study of PAAm hydrogels, they found that by increasing the proportion of ethanol in the polymerization, swelling of the synthesized hydrogels and their pore size increased. Random copolymer hydrogels of NIPAM and N-ethylacrylamide (NEAM) were prepared by Wang et al. using different proportions of methanol-water mixtures as synthesis-solvent [99]. The synthesis-solvent composition regulated the porosity (from non-porous to highly porous) of the hydrogels (Figure 3). The swelling ratio of the hydrogels also changed depending on the gel morphologies. High methanol concentration during hydrogel synthesis resulted in hydrogels with a higher swelling ratio due to a loosely connected network structure.

In a study by Zhang et al., a thermoresponsive PNIPA with a covalently incorporated crown ether derivative (4′-allyldibenzo-18-crown-6, CE) was copolymerized in a water–tetrahydrofuran (THF) mixed solvent [100]. The synthesis-solvent proportion in the mixed solvent during copolymerization affected the swelling properties of the synthesized PNIPA-co-CE hydrogels. The copolymer hydrogels showed faster deswelling rates than the normal PNIPA hydrogels at a high temperature (50 °C). They also found that hydrogels synthesized in a mixed solvent with lower THF content (33 vol%) exhibited a lower equilibrium swelling ratio (ESR). In contrast, hydrogels synthesized in a mixed solvent containing higher THF content (50 vol%) had a higher ESR value. This behavior is explained by the heterogeneity of the produced polymer network, which is in turn affected by the THF content in the synthesis-solvent mixture. Due to the lower polarity of THF compared to water, a mixed solvent with higher THF content produces hydrogels with a profoundly heterogeneous structure and enlarged polymer network.

In a recent study, crosslinked polyimide aerogels were synthesized using single or mixed solvents of DMF, N-methylpyrrolidone (NMP), and dimethylacetamide (DMAc) [101]. By changing the solvent or combination of solvents the polymer strand diameter, mesopore and macropore fractions, compressive modulus, and specific surface area of the aerogels were successfully manipulated. Using electron-donating solvents such as DMAc or NMP or including a block copolymer surfactant prolonged the gelation time. This resulted in the coarsening of the polymer strands and adversely affected the surface area and mesopore fraction. The shift from predominantly mesoporous to macroporous states due to prolonging gelation times also reduced the compressive modulus of the polyimide aerogels.

The free radical photopolymerization of N,N-dimethylacrylamide (NDMAm) using different solvents (i.e., water, ethylene glycol, methanol, THF, DMF, chloroform, and acetonitrile) was investigated by Valdebenito and Encinas [95]. They found that the polymerization rate increased by one order of magnitude when the synthesis-solvent shifted from an organic one to water. This enhancement was due to the strong hydrogen bond formation of the amide C=O group with water. Therefore, the chain transfer efficiency increased in water with respect to the organic protic solvents. This intermolecular hydrogen bonding of the chain transfer agents also affected properties such as molecular weight, polymer stereospecificity, phase transition, and the microgel behavior of poly(NDMAm). In another study, El-Halah et al. synthesized a series of polyacrylamide hydrogels by free radical polymerization using different solvents: water/ethanol (100/0, 80/20, 70/30, 60/40, 50/50, 40/60, 30/70, and 20/80 (V/V)) and water/dimethyl sulfoxide (DMSO) (100/0, 80/20, 70/30, 60/40, 50/50, 40/60, 30/70, and 20/80 (V/V)) [102]. The yields and swelling degree of the hydrogels were strongly affected by the selection of synthesis-solvent. Hydrogels synthesized in water/DMSO mixtures showed a higher swelling degree compared to those obtained using water/ethanol mixtures. The swelling degree of the hydrogels obtained in the water/ethanol mixture was directly related to the yield and pore size; this characteristic relationship was missing in hydrogels synthesized in water/DMSO mixtures. Moreover, in both solvent mixtures, the molar mass of the polymers and mechanical properties decreased as the water content in the solvent decreased. This decrease, however, was much more dramatic in ethanol than in DMSO. This behavior can be explained by the molecular size of the hydrogels, which were higher in hydrogels synthesized in water/DMSO than in hydrogels obtained in water/ethanol mixtures.

Synthesis of PNIPAm hydrogels by frontal polymerization using four mixed solvents (i.e., water/DMSO, ethanol/DMSO, THF/DMSO, and acetone/DMSO, respectively) was conducted by Feng et al. [103]. The solvent mixtures regulated the porosity and the swelling capacity of the synthesized hydrogels. Hydrogels synthesized in THF/DMSO had higher porosity as well as a higher swelling ratio. The lowest swelling ratio and lower porosity were obtained in hydrogels synthesized in the water/DMSO mixture.

Acrylamide (AAm) and N-2-hydroxyethylacrylamide (HEAAm) in combination with itaconic acid (IA) were used in one study to synthesize hydrogels using water and water/ethanol mixtures [104]. Although the physical properties of hydrogels synthesized in both synthesis-solvent media were similar, there were significant differences in the degree of swelling. Hydrogels synthesized in water/ethanol mixtures exhibited a higher swelling degree than hydrogels synthesized in pure water under the same reaction conditions.

In a recent study, polyacrylamide (PAAm) and poly(acrylamide-co-methyl methacrylate) (PAAm-co-MMA) hydrogels were synthesized in water, aqueous 1,4-dioxane (50 vol%) and aqueous ethanol (80 vol%) [105]. Morphological analysis indicated that the incorporation of MMA into the hydrogels was affected by the selection of synthesis -solvent. Moreover, hydrogels synthesized in aqueous ethanol displayed higher swelling values than the hydrogels synthesized in other solvents.

Recently, our group synthesized PNIPAm microgels via free radical polymerization using four solvents with different polarity index (PI), i.e., 1,4-dioxane, THF, toluene, and cyclohexane (PI: 5.2, 4.0, 2.4, and 0.2, respectively) [22]. Morphology and porosity analysis revealed that the microgels synthesized in polar solvents were smaller in size with smaller pore sizes, while those synthesized in non-polar solvents (i.e., toluene and cyclohexane) were larger with bigger pore sizes. Higher swelling capacity was observed in microgels synthesized in non-polar solvents, which was due to higher porosity in the gel structure. Due to the well-crosslinked network structure of the microgels synthesized in 1,4-dioxane and toluene, these microgels had better thermomechanical properties. This study confirmed that the porosity, swelling degree, and mechanical properties of the microgels can be tuned by choosing synthesis-solvents based on their polarity.

All these previous reports strongly indicate that the crosslinking in PNIPAm hydrogels is sensitive to the polymerization conditions, such as the synthesis-solvent. The reactivity of the solvent with the monomer, crosslinker, and initiator controls the crosslinking structure of PNIPAm. Tuning the hydrogel porosity, mechanical properties, and swelling behavior can be achieved by regulating the network structure formation during polymerization, which is in turn affected by the reactivity or polarity of the synthesis-solvent. Hence, it is crucial to gain a better understanding of the relationship between solvent reactivity and hydrogel properties. Establishing this relationship will enable the rational selection of synthesis-solvents for preparing PNIPAm-based hydrogel scaffolds with suitable properties for tissue engineering applications.

### 4.2. Effects of Crosslinking-Density

The addition of crosslinks between polymer chains influences the hydrogel’s physical properties, resulting in changes in elasticity, porosity, swelling, and degradability [106]. Interestingly, crosslinked PNIPAm-based hydrogels are unable to be dissolved in solvents, but can absorb large amounts of solvents [107,108,109]. Uncrosslinked PNIPAm is soluble in water while crosslinked PNIPAm is insoluble and absorbs water [107]. Porosity, mechanical properties, degree of swelling, and degradation behavior of PNIPAm hydrogels can be regulated by tuning the crosslinker content during synthesis.

#### 4.2.1. Phase Transition and Swelling Ability

**Figure 4:**
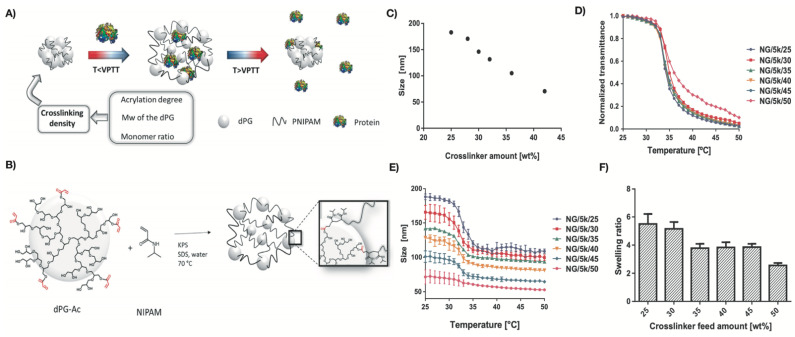
(**A**) Schematic outline of the temperature-triggered cargo release of dPG/PNIPAm nanogels, (**B**) synthesis outline of dPG/PNIPAm nanogels (dPG with average MW of 10 kDa), (**C**) size of the nanogels with different crosslinker content, (**D**) normalized transmittance curve of nanogels with different crosslinker content against temperature, (**E**) size-dependent transition of nanogels with changes in temperature, and (**F**) effect of the crosslinker content on the swelling ratios of the different thermoresponsive nanogels. Reproduced with permission from Navarro et al. (2020) [113].

The differences in the swelling behavior of PNIPAm hydrogels is caused by differences in crosslinking density, as the mesh size of the crosslinked hydrogel decreases as crosslinking density increases [110]. Mesh size is the average distance between crosslinks and corresponds to the pore size of the hydrogels [110]. The swelling ratio of hydrogels decreases by lowering the mesh size [111]. Therefore, crosslinking density has an inverse relationship with swelling ratio [112]: the higher the crosslinking density, the lower the swelling ratio, and vice versa. Swelling ability provides softness to the scaffolds and facilitates the diffusion of the nutrients, mimicking the biological environment and helping with tissue regeneration [11]. Navarro et al. recently prepared PNIPAm-based nanogels (NG) using dendritic polyglycerol (dPG) as a crosslinker and NIPAm as connecting thermoresponsive chains in different feed ratios (e.g., 30/70 wt%) [113]. Nanogels synthesized using lower content of NIPAm (NG/5k/50) were less thermoresponsive compared to others (Figure 4). This loss in temperature sensitivity was likely due to the induced rigidity of the network. Rigid polymeric networks were obtained due to the presence of higher amounts of dPG, which provided more crosslinking points, resulting in a denser network. Such a dense network contains shorter polymer chains between crosslinker points and decreases the flexibility of the PNIPAm-based nanogel structure. This also affected the swelling behavior of the nanogels; the swelling degree decreased as the dPG content increased. These results are also supported by Flory’s theory of elasticity. Nanogels with lower crosslinking density experience an abrupt phase transition due to lower elasticity components. The competition between elasticity and solvency is more pronounced, which results in an induced rigidity of the system. The increase in network density also decreases the nanogels’ pore size, enabling the retention of small molecular weight proteins. In another study, it was observed that when the stiffness of PNIPAm hydrogel particles decreased, the elastic plateau modulus became weaker in a dense PNIPAm suspension [114]. This was due to the higher crosslinking density of BIS (or MBAA), which led to a higher interparticle force. The authors also found a linear relationship between crosslinking density and the average hydrodynamic diameter of the hydrogel particles. It was observed that the particle sizes decreased with an increase in BIS content during synthesis.

**Figure 5:**
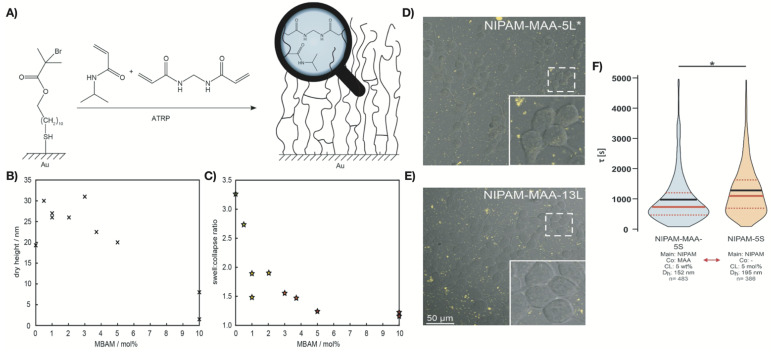
(**A**) Fabrication of thermoresponsive PNIPAm brush networks with MBAM in gold-coated ATRP initiator; (**B**) dry heights of PNIPAm networks decrease with increasing crosslinker content; (**C**) the swelling/collapsing height ratio in PNIPAm brushes reduces with increasing crosslinker content. Reproduced with permission from Thiele et al. (2021) [115]. (**D**,**E**) Confocal live-cell imaging of NIPAM-MAA-5L (**D**) and NIPAM-MAA-13L (**E**) microgels uptake in HEK293T cells. (**F**) Violin plots of uptake kinetics of microgels with co-monomer MAA (NIPAM-MAA-5S) (crosslinker: 5 wt%) and without MAA (NIPAM-5S) (crosslinker: 5 mol%). Dashed red lines show upper and lower quartiles. Reproduced with permission from Switacz et al. (2020) [116].

It is well established that the magnitude of the thermoresponsive transition of hydrogels can be regulated by introducing crosslinks between the polymer chains [115]. In a recent study, Thiele et al. tuned the thermoresponsive behavior of PNIPAm networks by varying the crosslinking density [115]. Different N,N’-methylene-bis-acrylamide (MBAM) contents (0 to 10 mol%) were evaluated for the synthesis of PNIPAm-co-MBAM networks via activators regenerated by electron transfer atom transfer radical polymerization (ARGET-ATRP) (Figure 5A–C). The swelling/collapse transition was least pronounced in hydrogel networks with a higher crosslinker content and was nonexistent in networks with 10 mol% MBAM. The collapse of the hydrogel network occurred within a broad temperature regime (from 27 to 34 °C) for all the MBAM contents investigated. The less crosslinked networks showed a sharp phase transition upon reswelling. Only PNIPAm-co-MBAM containing more than 3 mol% crosslinker exhibited a broadened transition. Such behavior persisted for PNIPAm-co-MBAM gels on nanostructured gold surfaces.

Thermoresponsive PNIPAm-based microgels were developed by Switacz et al. using NIPAM, comonomer MAA, and crosslinker MBAA with different contents (5 mol%, 10 mol%, 13 mol%, and 15 mol%, respectively) (Figure 5D–F) [116]. The hydrogels’ cellular uptake capacity and kinetics were affected by the extracellular microgel concentrations, resulting in gradients across the plasma membrane. Both microgel size and crosslinker content were found to affect the specific uptake kinetics in HEK293T cells. The combination of higher crosslinker content (>10 mol%) and relatively large microgel sizes (>800 nm) hindered cellular internalization. Significant cellular uptake was observed in smaller and softer PNIPAm microgels that translocated into the cytosol. This study was conducted at temperatures above the volume phase transition temperature (VPTT). Hence, caution is needed when conducting experiments and comparing with previous results due to the network elasticity effects of the temperature-induced collapse of microgels.

Burmistrova et al. synthesized PNIPAm-co-AAc hydrogels with three different MBAA contents (2%, 5%, and 10%) and analyzed the effect that crosslinker content had on the swelling/collapsing behavior and Young’s modulus [117]. The swelling ratio and hysteresis in the reversible swelling/collapsing decreased with increasing crosslinker content. The Young’s modulus, on the other hand, increased with increasing crosslinker content. The expected increase in swelling with decreasing MBAA incorporation was also observed by Mohapatra et al. in polystyrene-co-PNIPAm (PS-co-PNIPAm) microgels with different MBAA contents (0 to 3 mol%) [118]. In this study, the bulk modulus was found to be largely insensitive to crosslinking density while Young’s modulus was more sensitive to increased crosslinking density.

The softness, swellability, and deformability of PNIPAm-based microgels are regulated by crosslinking density, which in turn, controls the morphology and compressibility [119]. The deformability is controlled by the crosslinking ratio and increases with increasing swellability of the microgels [119]. PNIPAm-based microgels with different MBAA densities (1, 2.5, 5.0, 7.5, and 10 mol%, respectively) showed a crosslinking-dependent swelling at lower temperatures with the volume phase transition occurring at approximately 32 °C [119]. As expected, the swelling degree increased with decreasing crosslinking density. Mi et al. synthesized alginate-g-PNIPAm (APN) copolymeric hydrogels using PNIPAm-NH_2_, N-hydroxy succinimide (HOSu), N,N’-dicyclohexylcarbodiimide (DCC), and sodium alginate [120]. The swelling ratio of the APN hydrogels was inversely proportional to crosslinking density. Furthermore, higher crosslinking density resulted in APN hydrogels with smaller pore sizes, leading to a lower degree of release of blue dextran between 25 and 40 °C. Interestingly, the crosslinker density played an important role in controlling the porosity of PNIPAm-based nanocomposites. Carregal-Romero et al. showed that the porosity of a PNIPAm nanocomposite (Au@PNIPAm) decreased with increasing crosslinker concentration [121]. Jafari and Kaffashi synthesized dextran-hydroxymethyl methacrylate (Dex-HEMA) and PNIPAm copolymerized nanogels using different amounts of MBAA (0.25, 0.5, and 0.75 w/w%, respectively) via a solvent-free synthesis process [122]. The swelling ratio was affected by the crosslinking agent content; nanogels with the least amount of MBAA had the highest swelling ratio and water content. In addition, while the lower crosslinking density allowed the polymer chains to move more freely, the higher crosslinking density halted the chains from free movement due to their tighted arrangement. A similar relationship between swelling degree and crosslinking density was reported by Obeso-Vera et al. for PNIPAm microgels and copolymers synthesized using different crosslinker agents (i.e., MBAA, ethyleneglycoldimethacrylate, and EGDMA) [123]. Swelling is in general affected by the degree of crosslinking, the interfacial tension, and the particle size. In the study by Obeso-Vera et al., PNIPAm gel particles were nearly identical; therefore, the differences in crosslinking density were considered the primary factor determining the changes in swelling ratios.

#### 4.2.2. Mechanical Strength

The mechanical behavior of the hydrogel is strongly dependent on the crosslinking density [124]. As crosslinking density decreases, the elastic modulus decreases. Tuning the mechanical properties is crucial as hydrogel scaffolds for in vivo cartilage regeneration should have excellent strength and elasticity to support stretching, bending, and friction [125]. Porosity also has effects on the mechanical properties and in vitro degradation of the hydrogel scaffolds [126]. It is evidenced that the porosity of the hydrogel increases with decreasing crosslinking density [127]. Upon implantation, local angiogenesis occurs with the help of the porosity of hydrogels [128]. Scaffold porosity is also responsible for facilitating cell survival and proliferation [129]. The extent of secretion of ECM also is enhanced by increasing porosity [128,130]. Tan et al. incorporated different concentrations of starch-based nanospheres (SNs) (0.25 to 1.0 g/g of NIPAM) into the structure of thermoresponsive PNIPAm hydrogels (TPHs) [131]. In general, the mechanical strength of TPHs increased with increasing SN content. Hydrogels with 0.75 g of SN provided the maximum strength at 8.44 MPa; further increase in SN content, however, led to a sharp reduction in Young’s modulus, as the aggregation of superfluous SNs acted as structural defects instead of increasing the crosslinking in the hydrogels. In addition, the strain to failure of TPHs showed a tendency to decrease with increasing crosslinking density. Lehmann et al. synthesized PNIPAm hydrogels using MBAA (or BIS) and poly(ethylene glycol)diacrylate (PEGDA) as crosslinkers [132]. A decrease in crosslinking density increased the mesh size, which enabled the P(NIPAm-PEGDA) hydrogels to release larger volumes of water from the network compared to conventional PNIPAm hydrogels P(NIPAm-BIS). Moreover, a higher storage modulus (G’) independent of temperature could be obtained by increasing the crosslinking density of both P(NIPAm-PEGDA) and P(NIPAm-BIS) hydrogels. The difference in the viscoelastic response of the P(NIPAm-PEGDA) hydrogels increased with decreasing crosslinking density (increasing deswelling ratio). The highest storage modulus was obtained in hydrogels with the lowest crosslinking density (up to a 50-fold increase in G’).

Hydrogels crosslinked with metals have also been explored to enhance the mechanical strength of hydrogel scaffolds. Metal-crosslinked hydrogels can be obtained via metal coordination and covalent crosslinking [133,134]. Recently, a hybrid hydrogel was fabricated through metal coordination between ferric ions (Fe^3+^) and carboxyl groups of poly(acrylamide-co-acrylic acid) (P(AAm-co-AAc) [135]. This physically crosslinked hydrogel provided higher stiffness and toughness, resistance to fatigue, shape-memory ability, and stimuli-responsive healing. In another study, iron-containing diblock copolymer poly[N-isopropylacrylamide-co-2-nitrobenzyl acrylate)-block-(N,N-dimethylacrylamide-co-acrylic acid) hydrogels were prepared via coordination interaction between Fe^3+^ and carboxylates of polymers [136]. Such amphiphilic hydrogels showed better mechanical strength and exhibited sol–gel transition in response to different stimuli (i.e., UV, multidentate ligand, and redox agent Na_2_S_2_O_4_). Previously, Andzelm et al. produced hydrogels with enhanced storage moduli by adding divalent or trivalent ions (Zn^2+^, Al^3+^, Ca^2+^, Cu^2+^, and Fe^3+^) into cellulose nanofibril dispersions [137]. In a recent study, an Fe-containing hydrogel was formed by one-pot free radical polymerization of acrylic acid with MBAA as covalent crosslinker and Fe(NO_3_)_3_ as the ionic crosslinker [138]. Such a covalently crosslinked structure made the PAA hydrogels mechanically superior (high toughness) and stretchable. It was reported earlier that Cu^2+^, Zn^2+^, and Fe^3+^ could act as noncovalent crosslinkers in hybrid polymer synthesis [139]. Makris et al. showed that the presence of copper sulphate enhanced the activity of lysyl oxidase (LOX) to form collagen crosslinks, resulting in an increase in the integrity and strength of neo-cartilage [140]. Some studies demonstrated the efficiency of copper in promoting chondrocyte proliferation, differentiation, and cartilage matrix generation by enhancing secretions of insulin like growth factor-1 (IGF-1), IGF-binding protein-3, and transforming growth factor-β (TGF-β) [141,142]. Although metals can be used as crosslinkers to form metal-containing hybrid hydrogel scaffold for cartilage tissue engineering, the complexity in terms of preparation processes is still a big challenge. For example, copper can induce the crosslinking of collagen to change the mechanical properties during cartilage regeneration, but interaction between copper and base materials (e.g., NIPAm) is still a major concern [143]; it is difficult to control the rate of crosslinking and crosslinking structure between copper and the base materials [143]. Hence, systematic research should be performed in future to understand the influence of metals in chondrogenesis and determine the appropriate use of metals as crosslinkers in metal-based hydrogel scaffold synthesis for cartilage regeneration.

Swelling degree, porosity, and mechanical strength are some key properties that are needed in PNIPAm-based scaffold materials for the regeneration of tissues including cartilage. Crosslinking density regulates all these properties. Porosity and crosslinking density have a linear relationship while swelling degree has a reversible relationship with crosslinking density. The degree of porosity or mesh size has a direct effect on the mechanical strength or stiffness of the scaffold [129]. The stiffness of the scaffold increases when porosity decreases and vice versa [144]. Therefore, porosity, swelling degree, stiffness, and crosslinking density are correlated with each other. Hence, tuning the crosslinking density during synthesis to prepare PNIPAm-based hydrogel scaffolds with required properties will help to make them ideally suited for cartilage or muscle tissue engineering applications.

## 5. Challenges and Future Perspectives in Tuning Scaffolds Properties

**Figure 6:**
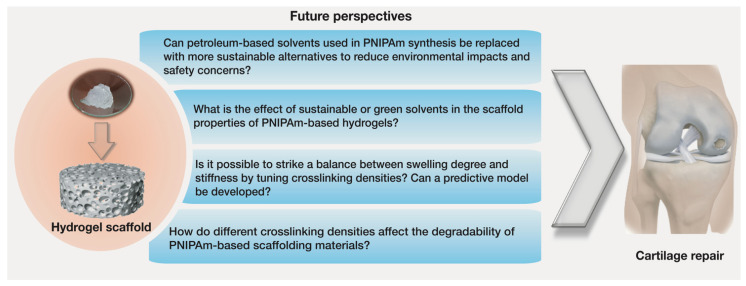
Future research questions need to be addressed to improve the synthesis and scaffolding properties of PNIPAm-based hydrogels for cartilage tissue engineering.

Many synthesis methods and hydrogel scaffolds with functional advantages have been developed for tissue engineering applications [8,11]. Still, designing a fully tunable scaffold with physicochemical properties similar to those of the cartilage tissue is the major outstanding challenge. Additional studies are needed to identify the design parameters that will result in PNIPAm-based scaffolds with biomimetic structures and improved biochemical and biophysical properties more suitable for cartilage tissue repair (Figure 6). Moreover, there is also a need to improve the safety and sustainability of the synthesis methods.

### 5.1. Sustainable Synthesis Methods

The synthesis of PNIPAm hydrogels is commonly performed via free radical polymerization [22], requiring NIPAm monomer, crosslinker, initiator, and a synthesis-solvent. The synthesis-solvent has been found to play a crucial role in tuning some of the key properties required for cartilage tissue engineering [11,22,145]. In particular, the polarity of the synthesis-solvent affects its reactivity and the physicochemical and mechanical properties of synthesized hydrogels. The solvents that have been used in PNIPAm synthesis are unfortunately not environmentally benign [146]. Petroleum-derived solvents, such as THF, toluene, and 1,4-dioxane, are more frequently used as they are inexpensive [147]. However, these solvents are known hazardous air pollutants, reprotoxins, carcinogens, and mutagens [148]. Moreover, most of these solvents are also volatile and flammable [149,150]. Although high volatility enables easy recovery and purification via distillation, it also increases the likelihood of toxic air emissions and the risk of exposure faced by workers [148]. In addition, energy-intensive separation and purification methods are required to ensure the complete elimination of the solvent from the final hydrogel scaffold [151]. As safety and environmental concerns become more dominant in the material synthesis industry, there is a need to investigate alternative solvents for the sustainable production of hydrogel scaffolds.

Water is considered a green solvent for sustainable synthesis processes. There are reports on the synthesis of PNIPAm hydrogels using water as synthesis-solvent [96,105]. However, the response rate of hydrogels synthesized in water is low due to the formation of a dense skin layer in the collapsed gels within the initial 30 min of shrinking [152]. Such behavior hinders the mass transport of water out of the hydrogels and limits their application in the field of tissue engineering. Moreover, after synthesis, recovery of wastewater via distillation or other purification processes is needed, which can be energy-intensive and also reduces the sustainability of using water for the polymer synthesis process [153].

Among the other suggested solvents in the last few years for the sustainable synthesis of polymers are ionic liquids (ILs), monoterpenes (MTs), and supercritical fluids (e.g., supercritical carbon dioxide). [146,148,149,150,154,155,156]. These environment-friendly solvents have been reported as synthesis-solvents for different polymeric materials. Some of these sustainable solvents show performances during free radical polymerization comparable to conventional petroleum-based solvents. However, there is an urgent need to investigate the effect that these sustainable solvents have on the quality and properties of the PNIPAm-based scaffold materials for tissue engineering.

### 5.2. Biodegradability

Despite the advantages of PNIPAm-based scaffolds for tissue engineering applications, the nonbiodegradability of PNIPAm is a lingering concern [69]. Although partial or completely degradable PNIPAm-based copolymer scaffold materials have been developed, there are still some gaps in this area and more efforts are needed. For example, premature degradation of scaffolds induces vascularization and triggers premature ingrowth of peri-implantation of tissue into the central region of the scaffold [157]. Hence, controlled degradation of scaffolds is crucial for the complete regeneration of tissue. Crosslinking density can be an option to tune the degradability of PNIPAm-based scaffolding materials to a relevant time frame for tissue repair [59]. Porosity also has an impact on the degradability of hydrogels; the higher the porosity, the greater the degradation rate. Tuning the porosity in PNIPAm hydrogels can be achieved by adjusting the crosslinking density.

### 5.3. Stiffness, and Swelling Degree

Better degrees of swelling and higher mechanical strength are both desired for tissue engineering applications [158]. It is well established that the swelling degree has an inverse relationship with the stiffness of the hydrogel materials [159]. If it could be possible to tune the crosslinking structure of PNIPAm-based materials to control both swelling degree and stiffness, then these materials would emerge as a preferred scaffolding material for tissue regeneration. Apart from tuning the crosslinking structure, another way to enhance the stiffness of the scaffold is via 3D or 4D printing. Although 4D printing uses the same manufacturing processes used in 3D printing, the main difference is the type of the materials used [160]. The materials for 4D printing exhibit a smart behavior upon exposure to an external stimulus such as temperature, light, or pH [160]. Therefore, the final 4D printed product can change its shape, function, or other physicochemical properties in response to the above-mentioned stimuli. Smart materials such as PNIPAm can be used to make 4D scaffolds with tunable stiffness and well-defined internal organization to adapt to their microenvironment [160]. However, the reversible thermoresponsive feature of PNIPAm-based scaffolds has some drawbacks during printing: impaired printability of the material and lack of desired 4D effects in the product [161]. Moreover, the presence of interfacial defects in the 3D layered structure (in layer-by-layer printing) of polymer-based scaffolds is another major pitfall [162,163]. This drawback leads to the poor stiffness in the final 3D printed scaffold. These pitfalls could be resolved by using stimuli-responsive polymers in combination with different concentrations of non-responsive crosslinkers or polymers, which would serve as a mechanical property enhancer. This would lead to the synthesis of stiffer 3D or 4D printed PNIPAm-based scaffolds for cartilage repair.

## 6. Concluding Remarks

Thermoresponsive PNIPAm-based hydrogel scaffolds with modified structures and physicochemical properties are critical for cell function in cartilage tissue engineering. Various design variables including choice of synthesis-solvent and crosslinking density during polymerization can alter the structure and properties of PNIPAm scaffolds. Such alterations make the microenvironments of the scaffolds more favorable for tissue regeneration. In this review, we presented a comprehensive overview of the key properties of scaffolds and the effects that synthesis-solvent and crosslinking density have on tuning these properties. Although the unique properties of PNIPAm-based hydrogels make them a potential candidate for cartilage tissue repair application, further research is needed to further enhance their mechanical properties and biodegradability. So far, several PNIPAm-based scaffolding materials have shown improved structural properties and physicochemical behavior, but they still do not match all the functions of the in vivo microenvironments. PNIPAm-based scaffolds with controlled architecture must be designed and studied to assess their feasibility for clinically viable cartilage tissue regeneration. As highlighted in Section 5 and Figure 6, future research should focus on developing more sustainable synthesis pathways, designing advanced procedures to synthesize hydrogel scaffolds with optimized architectures, and generating accurate models to predict the properties of hydrogels so as to enable the optimization of synthesis conditions to obtain better scaffolding performance.

## Data Availability

Not applicable.
